# Correlation of Salivary Statherin and Calcium Levels with Dental Calculus Formation: A Preliminary Study

**DOI:** 10.1155/2017/2857629

**Published:** 2017-05-10

**Authors:** Deepak Gowda Sadashivappa Pateel, Shilpa Gunjal, Swarna Y. Math, Devarasa Giriyapura Murugeshappa, Sreejith Muraleedharan Nair

**Affiliations:** ^1^Oral Pathology and Oral Medicine, Faculty of Dentistry, MAHSA University, Bandar Saujana Putra, 41200 Jenjarom, Selangor, Malaysia; ^2^Department of Dental Public Health, Faculty of Dentistry, MAHSA University, Bandar Saujana Putra, 41200 Jenjarom, Selangor, Malaysia; ^3^Oral Diagnostic and Surgical Science Division, International Medical University, No. 126, Jalan 19/155B, 57000 Bukit Jalil, Kuala Lumpur, Malaysia; ^4^Department of Pedodontics, Faculty of Dentistry, MAHSA University, Bandar Saujana Putra, 41200 Jenjarom, Selangor, Malaysia; ^5^Biomedical Sciences, MAHSA University, Bandar Saujana Putra, 41200 Jenjarom, Selangor, Malaysia

## Abstract

**Background:**

Salivary constituents have a wide range of functions including oral calcium homeostasis. Salivary proteins such as statherin inhibit crystal growth of calcium phosphate in supersaturated solutions and interact with several oral bacteria to adsorb on hydroxyapatite. Concurrently, saliva, which is supersaturated with respect to calcium phosphates, is the driving force for plaque mineralization and formation of calculus. Thus, the aim of the present study was to estimate and correlate salivary statherin and calcium concentration to the dental calculus formation.

**Methods:**

A cross-sectional study was conducted to assess the relationship between salivary statherin, calcium, and dental calculus among 70 subjects, aged 20–55 years. Subjects were divided into 3 groups based on the calculus scores as interpreted by Calculus Index which was followed by collection of whole saliva using Super•SAL™. Salivary calcium levels were assessed by calorimetric method using Calcium Assay kit (Cayman Chemical, Michigan, USA) and statherin levels by using ELISA Kit (Cusabio Biotech).

**Results:**

Statherin levels showed a weak negative correlation with the calcium levels and with calculus formation. The mean salivary statherin and calcium concentration were found to be 0.96 *μ*g/ml and 3.87 mg/ml, respectively. Salivary statherin levels differed significantly among the three groups (*p* < 0.05).

**Conclusions:**

Our preliminary data indicates that statherin could possibly play a role in the formation of dental calculus.

## 1. Introduction

Saliva is essential for lifelong maintenance of oral health. Saliva is composed of a variety of electrolytes, immunoglobulins, proteins, and enzymes and plays some important functions in the maintenance of oral health such as lubrication of the oral mucosa, defence from infections, protection against demineralization [1].

The four major salivary proteins, that is, statherin, the acidic PRPs, cystatins, and histatins, are primarily responsible for the maintenance of the homeostasis of the supersaturated state of saliva with respect to calcium phosphate salts. Statherin is a very potent inhibitor of crystal growth in comparison with other salivary proteins as it has been shown to exhibit unusually high affinities for hydroxyapatite. It is a multifunctional peptide that possesses a high affinity for calcium phosphate minerals, maintains the appropriate mineral solution dynamics of enamel, promotes selective initial bacterial colonization of enamel, and functions as a boundary lubricant on the enamel surface. Statherin inhibits both nucleation and growth of hydroxyapatite crystal and its concentration. It is the only salivary protein that inhibits the spontaneous precipitation of calcium phosphate salts from the supersaturated saliva. It inhibits primary as well as secondary precipitation of calcium phosphate salts [2]. In addition, statherin may function in the transport of calcium and phosphate during secretion of salivary glands. Statherin concentration is not subject to circadian rhythms unlike other salivary peptides [3]. Besides that, statherin promotes bacterial adhesion to enamel surfaces, although weakly compared with other salivary macromolecules [4], and acts as a boundary lubricant at the enamel interface [5].

Precipitation of calcium from saliva is prerequisite for the formation of dental calculus. At the same time saliva as a defence system has proteins like statherin which inhibits this precipitation. Salivary statherin is believed to play a protective role and provides a stable environment for teeth. But little is known of its variations in occurrence or concentration in individual saliva samples of dental calculus formers. At present, the relationship between statherin and formation of dental calculus is unclear.

The aim of the present study was to estimate and correlate salivary statherin and calcium concentration to the dental calculus formation.

## 2. Methodology

The present cross-sectional study comprised of subjects aged between 20 and 55 years with different grades of calculus formation recruited from outpatients of Faculty of Dentistry, MAHSA University, Malaysia. Subjects with systemic diseases especially diabetes mellitus, salivary gland, thyroid and parathyroid gland pathologies, altered vitamin D metabolism, and hormonal disturbances affecting calcium metabolism and who are on *β*-adrenolytic drugs were excluded from the study. The study protocol and ethical clearance was approved by Research Review Committee, MAHSA University.

Based on the results of Al-Rafdain [6] study, a sample of 72 was calculated using the software Open Epi version 2.3. The power of the study was set at 80% and *p* < 0.05 as statistically significant.

Consecutive sampling technique was employed to recruit a total of 70 subjects meeting the inclusion and exclusion criteria. A brief dental history and oral examination was performed and recorded along with the demographic data. The amount of calculus formation was recorded as per the calculus component of Oral Hygiene Index [7] followed by collection of saliva samples.

The selected subjects were divided into three groups based on the calculus scores as follows: Group I (low calculus group): calculus score of 0–0.6; Group II (medium calculus group): calculus score 0.7–1.8; Group III (high calculus group): calculus score 1.9–3.

*Saliva Collection*. Super•SAL, Oasis Diagnostics®, saliva collection kit was used for the collection of whole mouth saliva from the study subjects. Subjects were advised not to eat or drink anything 30 minutes prior to saliva collection. Microfuge tube was attached to the plastic compression tube firmly and placed on a sterile area. White absorbent collection pad end of the Super•SAL device was placed along the side of the tongue in an upright position in the mouth until the volume adequacy indicator changes from yellow-green to blue. Absorbent pad end was removed from oral cavity and placed into the plastic compression tube and plunger was pushed downwards to transfer saliva to a microfuge tube. The microfuge tube was removed from the device. The lid of the microfuge was closed and it was stored at −70°C until further analysis. Salivary samples were centrifuged at 4000 rpm using centrifuge. Each sample was assessed for statherin and calcium levels. Calcium levels were assessed by calorimetric method using Calcium Assay kit (Cayman Chemical, Michigan, USA) and statherin levels by using ELISA Kit (Cusabio Biotech). All the reagents were of analytical grade. Concentrations of calcium and statherin were estimated by spectrophotometer and calculated by measuring optical density of 575 nm and 450 nm, respectively. Two samples were omitted because of technical error during ELISA procedure.

The data was analyzed using SPSS statistical package (version 20.0 SPSS Inc., Chicago, IL, USA). Differences between mean calcium and statherin levels between three groups were assessed using Kruskal-Wallis test. Spearman's correlation coefficient was used to correlate between statherin, calcium, and calculus levels. *p* value < 0.05 was considered statistically significant.

## 3. Results

A study group is comprised of 42% (29) males and 58% (41) females. The mean salivary calcium concentration and statherin and calculus scores among the study subjects were 3.87 mg/ml, 0.96 *μ*g/ml, and 1.35, respectively.

In the present study, weak negative correlation was observed when statherin, calcium, and calculus levels were correlated which was statistically nonsignificant (*r* < 0.2,  *p* > 0.05) (Table 1). The difference between salivary statherin levels among three groups was found to be statistically significant (*p* = 0.04). However, differences between salivary calcium levels among three groups were not statistically significant (*p* > 0.05) (Table 2).

## 4. Discussion

Excess salivary calcium has been reported in patients with tendency to develop supra- or subgingival calculus [6] and supersaturation of saliva with respect to calcium phosphate salts is the driving force of calculus formation [8]. In the present study the mean salivary calcium concentration was found to be 3.87 mg/ml and had a positive correlation with the amount of calculus formation in accordance with the previous studies. However, this correlation was statistically insignificant (*p* = 0.71). Diverse salivary calcium levels reported in numerous studies (4–6 mg/dL, 0.5–1.5 mmol/L, and 1–4 mmol/L [9, 10]) are probably due to the different methodologies employed.

In the present study, the salivary statherin concentrations were estimated and were found to be ranging from 0.5 to 4.0 *μ*g/ml. The previous studies on statherin have employed different methods for estimation of its concentration which explains the varied concentrations of statherin reported in numerous studies [11, 12]. In the present study salivary statherin demonstrated a statistically significant difference (*p* = 0.004) among the selected groups with different grades of dental calculus formation.

Experiments have shown that statherin has a critical role to play with respect to calcium phosphate homeostasis in the oral environment. It is explained that statherin exhibits adsorption associated folding that is related to its particular functions both off and on the surface. This significant structural transition is coupled with the key functions that statherin exhibits, including the binding of early crystal nuclei to suppress the onset of calcium phosphate crystallization and adsorption onto nucleated crystals to inhibit their growth [5, 11]. The compact fold of statherin guarantees efficient coverage of the crystal faces and effective masking of charged residues that may otherwise promote formation of new crystal layers [13]. This significant structural transition is coupled to the key functions that statherin exhibits, including the binding of early crystal nuclei to suppress the onset of calcium phosphate crystallization and adsorption onto nucleated crystals to inhibit their growth [14].

Statherin in pellicle is distributed close to the enamel surface and has a strong calcium binding activity [15, 16]. However, a recent study has shown that a statherin- and calcium-rich layer also forms at the air-liquid interface of salivary films in vitro [17]. Because of these observed properties, statherin is thought to be a major regulator of mineralization in the mouth. In the first of the study to compare and correlate the relation between salivary calcium and statherin levels with the amount of dental calculus formation, a weak negative correlation was observed between salivary statherin, salivary calcium levels, and calculus score (*r* < −0.2). Salivary statherin is a multifactorial protein and performs multiple functions [18]. Whether its concentrations are influenced by other factors like microbial load and demineralization needs to be further investigated.

## 5. Conclusion

The present study is a valiant attempt which highlights the possible potential relationship between the salivary statherin and dental calculus. Recently there has been an increasing interest in identification of disease specific salivary markers with clinical relevance. The observations from the present preliminary study indicate the following:

- Salivary statherin levels differ significantly with different grades of calculus.
- Salivary statherin is inversely proportional to the calculus deposition.

Statherin could possibly play a role in the formation of dental calculus formation. However, the conclusions obtained should be generalized with caution due to smaller sample size.

## Figures and Tables

**Table 1:** Spearman's correlation between salivary statherin level, salivary calcium level, and calculus score.

	Calcium levels (mg/ml)	Statherin levels (*µ*g/ml)	Calculus score
Calcium levels (mg/ml)			
Spearman's rho	1.000	−0.088	−0.075
*p* value	—	0.46 (NS)	0.53 (NS)
Statherin levels (*µ*g/ml)			
Spearman's rho	−0.088	1.000	−0.093
*p* value	0.46 (NS)	—	0.44 (NS)

*p* > 0.05 nonsignificant (NS).

**Table 2:** Comparison of salivary calcium and statherin levels based on the calculus scores.

Calculus score	*N*	Mean ± Sd	
		Salivary calcium (mg/ml)	Salivary statherin (*µ*g/ml)
Group I (low calculus group)	16	3.44 ± 0.44	1.02 ± 0.99
Group II (medium calculus group)	32	3.76 ± 3.33	1.01 ± 0.80
Group III (high Calculus group)	22	6.12 ± 1.30	0.85 ± 0.97
*p*-value		0.71 (NS)	0.04^*∗*^

^*∗*^ *p* < 0.05 statistically significant; applied Kruskal-Wallis test; *p* > 0.05 nonsignificant (NS).

## Additional Points

*Limitations of Present Study and Future Directions.* (i) Studies with larger sample size are required to further substantiate the results of the present study. (ii) The influence of bacterial proteases and salivary flow on the statherin levels should be investigated using both whole mouth saliva and ductal saliva. (iii) Both free and protein-bound calcium need to be investigated. (iv) Longitudinal follow-up studies to investigate the long-term influence of the statherin and other proteins related to calcium hemostasis, like PRPs and cystatins, on oral hygiene status, caries, erosion, and dental calculus formation in particular are required.
